# Albumin-based nanosystem for dual-modality imaging-guided chem-phototherapy against immune-cold triple-negative breast cancer

**DOI:** 10.1093/rb/rbad073

**Published:** 2023-08-31

**Authors:** Chen Peng, Xiaodie Zeng, Jiali Cai, Hanyu Huang, Fan Yang, Shaowen Jin, Xiuhong Guan, Zhiyong Wang

**Affiliations:** Key Laboratory for Polymeric Composite and Functional Materials of Ministry of Education, School of Materials Science and Engineering, Sun Yat-sen University, Guangzhou 510275, China; Key Laboratory for Polymeric Composite and Functional Materials of Ministry of Education, School of Materials Science and Engineering, Sun Yat-sen University, Guangzhou 510275, China; Key Laboratory for Polymeric Composite and Functional Materials of Ministry of Education, School of Materials Science and Engineering, Sun Yat-sen University, Guangzhou 510275, China; Key Laboratory for Polymeric Composite and Functional Materials of Ministry of Education, School of Materials Science and Engineering, Sun Yat-sen University, Guangzhou 510275, China; Department of Pediatrics, Department of Nuclear Medicine, Guangdong Provincial Engineering Research Center of Molecular Imaging, The Fifth Affiliated Hospital, Sun Yat-sen University, Zhuhai 519000, China; Department of Gastrointestinal Surgery, Sun Yat-sen Memorial Hospital, Sun Yat-sen University, Guangzhou 510120, China; Department of Radiology, The Sixth Affiliated Hospital of Guangzhou Medical University, Qingyuan People’s Hospital, Qingyuan 511518, China; Key Laboratory for Polymeric Composite and Functional Materials of Ministry of Education, School of Materials Science and Engineering, Sun Yat-sen University, Guangzhou 510275, China

**Keywords:** albumin, magnetic resonance imaging, fluorescence near-infrared II imaging, phototherapy, nanozyme

## Abstract

Triple-negative breast cancer is a highly aggressive and metastatic tumor; diagnosing it in the early stages is still difficult, and the prognosis for conventional radio-chemotherapy and immunotreatment is not promising due to cancer’s immunosuppressive microenvironment. The utilization of protein-based nanosystem has proven to be effective in delivering agents with limited adverse effects, yet the combination of diagnosis and treatment remains a difficult challenge. This research took advantage of natural albumin and organic molecules to construct a self-assemble core-shell nanostructure combining with superparamagnetic iron oxide nanocrystals and heptamethine cyanine dye IR780 through non-covalent interactions. This nanocomposite successfully decreased the transverse relaxation time of the magnetic resonance hydrogen nucleus, resulting in outstanding *T*_2_ imaging, as well as emitting near-infrared II fluorescence, thereby the resulting dual-modality imaging tool was applied to improve diagnostic competency. It is noteworthy that the nanocomposites exhibited impressive enzyme-like catalytic and photothermal capabilities, resulting in a successful activation of the immune system to efficiently suppress distant metastatic lesions *in vivo*. Consequently, this nano-drug-based therapy could be an advantageous asset in reinforcing the immune system and hindering the growth and reappearance of the immune-cold breast cancer.

## Graphical Abstract

**Figure:**
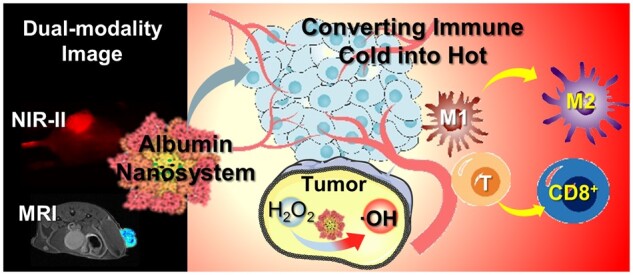

## Introduction

Triple-negative breast cancer (TNBC) is an aggressive subtype of breast cancer that lacks estrogen receptor, progesterone receptor and human epidermal growth factor receptor-2 receptors [1]. Currently, surgery, radio-chemotherapy and hormone therapy remain the predominant treatment option [2]. However, the prognosis of patients suffering from TNBC is still unsatisfactory due to the special inflammatory-tumor-microenvironment, which leads to a diminished immune response, a desirable therapeutic efficacy and high risk of recurrence [3]. To address these challenges, new medicinal strategies are expected to improve the outcomes of this ‘immune-cold’ cancer [4]. Phototherapy, a non-invasive cancer treatment that harnesses external light energy to generate heat with the assistant of photo-absorbing agents, has the potential to provide precise treatment and stimulate an immune response, resulting in reduced tumor resistance [5, 6]. Accurate identification of the intended areas is of utmost importance when applying photothermal treatment, as this will enhance the therapeutic effects while reducing harmful side effects [7]. Therefore, guidance from clinical imaging is necessary for this strategy; and the application of contrast agents could effectively increase imaging sensitivity, allowing for a precise diagnosis. Moreover, integrating imaging agents with therapeutic compounds could develop a theranostic platform with a great potential for real-time monitoring of the treatment process and feedback on the effectiveness of therapy and this approach would enable clinicians to tailor therapies according to individual patient needs, improving outcomes and minimizing the risk of adverse events [8].

Magnetic resonance imaging (MRI) is a non-invasive clinical imaging technique that has proven valuable in obtaining bioinformation by measuring relaxation time of hydrogen protons [9]. The utilization of superparamagnetic iron oxide (SPIO) contrast agent has been demonstrated to significantly increase imaging sensitivity and plays an important role in preoperative diagnosis [10]. In addition, recent study revealed that these nanocrystals were able to perform enzyme-like catalytic reaction with hydrogen peroxide (H_2_O_2_) [11]. Given a high level of H_2_O_2_ in tumor microenvironment, these SPIO agents can be used to catalyze H_2_O_2_ to generate ROS for cancer cell killing to improve the therapeutic effect [12]. Hence, the combination of SPIO and MRI technology enables the integration of imaging and therapy, although the harsh operating condition of MRI make it difficult to perform intraoperative navigation. Near-infrared II (NIR-II) fluorescence imaging with emission wavelengths from 1000 to 1700 nm, provides a high resolution image of tissue with minimal scattering [13]. Under near-infrared light irradiation, the heptamethyl cyanine dye IR780 displays strong absorption ability, efficient fluorescence, remarkable photothermal conversion efficiency, and is suitable for optical imaging and photothermal therapy [14]. While optical imaging is advantageous for real-time monitoring during interventions, deep imaging is intrinsically challenging within the body [15]. Hence, merging optical imaging and MRI modalities could counteract the respective drawback and achieve maximum detection benefit; and the combination of multimodal contrast agents and therapeutic medications could be employed to maximize the treatment outcome.

The nanostructured system has the potential to meet a variety of needs in biomedical research and development [16]. Serum albumin, a biomacromolecule with excellent biocompatibility, possesses hydrophilic and hydrophobic regions and contains various chemical elements like amino and carboxyl group, giving it a unique bifunctional domain [17]. Covalent bond connecting techniques have been employed to formulate albumin-based nanovehicle [18]. Despite this, these chemical conjugations easily alter the protein functional regions, leading to biosafety concerns [19]. Thereby, developing an uncomplicated and environmentally friendly method for nanoplatform construction remains a significant challenge. In this study, we established a self-assembly method of albumins with small molecules to encapsulate IR780 and SPIO forming a nanosystem, IR780@BSA@SPIO. With the NIR-II and MRI dual-mode imaging, this nanosystem has the capability to accurately locate cancer and guides photothermal therapy to efficiently eliminate tumors. Additionally, the nanosystem stimulates the immune system and effectively impedes the spread of metastatic triple-negative breast tumors, even in more remote regions. Our results demonstrate the potential of our nanosystem for biomedical applications.

## Results and discussion

### Preparation and characterization of the albumin-based nanosystem

**Figure 1.**
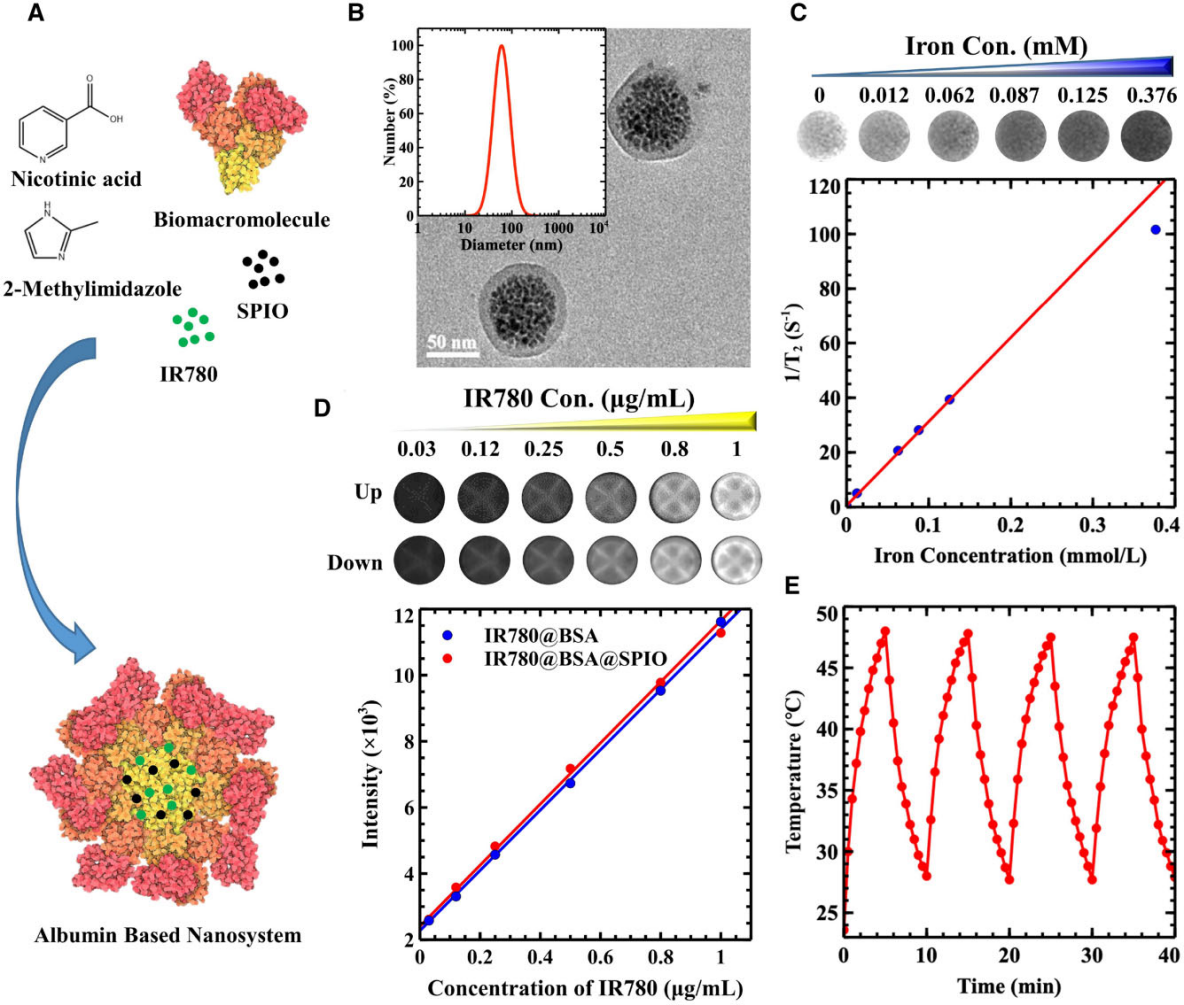
(**A**) Synthesis diagram of IR780@BSA@SPIO nanosystem. (**B**) DLS data and TEM characterization of IR780@BSA@SPIO nanosystem. (**C**) T_2_WI image and the relationship between the relaxation value and concentration of IR780@BSA@SPIO nanosystem. (**D**) NIR-II fluorescence imaging of IR780@BSA@SPIO and IR780@BSA two systems, and the relationship between the signal intensity and IR780’s concentration. The image on the top shows the fluorescence image of IR780@BSA while the one at the bottom displays the fluorescence image of IR780@BSA@SPIO. (**E**) Heating absorption and dissipation rocking result of nanosystem under 808 nm laser irradiation at [IR780] 1.5 μg/mL.

SPIO exhibits superior magnetic characteristics and is commonly applied as contrast agent in medical MRI [20]. The IR780 organic small molecule has been widely noticed for its ability to produce a NIR-II fluorescence imaging as well as photothermal conversion effect, making it a promising candidate for optical imaging and photothermal therapy [21]. Although SPIO crystals of high quality can be synthesized through thermal decomposition, the presence of hydrophobic surfactants substantially restricts their use in the medical field [22]. The application of IR780 molecule also experiences similar issues, and its indole structure causes low solubility in water [23]. Albumin possesses amphiphilic functional groups, enabling it to form nanostructures in a watery environment [24]. Our prior study demonstrated that nicotinic acid and 2-methylimidazole create a functional domain through the hydrophobic and pi–pi interaction. Additionally, the carboxyl group of nicotinic acid, as well as the nitrogen atom of imidazole molecule enable the creation of hydrogen bonds. Therefore, this structure will be linked to the bovine serum albumin (BSA) protein through non-covalent interactions, improving the stability of the nanosystem [25]. At the same time IR780/SPIO can be encapsulated by the biological macromolecule BSA through hydrophobic interactions, as well as the π–π stacking interactions with amphiphilic small molecules, such as nicotinic acid and dimethyl imidazole [26]. Critical micelle concentration (CMC) is a crucial factor in determining the ability of surfactant molecules to form micelles in a solvent [27], and the value of the free BSA sample was calculated to be 29.51 g/L, while the BSA-based nanosystem showed a result of 13.80 g/L in Supplementary Figure S1. The results suggested that the small molecules, nicotinic acid and dimethylimidazole, could keep the nanostructure stable at lower concentrations than that of natural free BSA. Afterwards, a core-shell nanosystem consisting of BSA encapsulating hydrophobic IR780 and SPIO nanocrystals was prepared (Figure 1A), forming IR780@BSA@SPIO with highly package efficiency of 80% and 74% for the IR780 and SPIO cargoes, respectively. Moreover, the nanoparticle’s dimension and morphology were evaluated in Figure 1B. Here, the particles showed a diameter of ∼60 nm, and were evenly distributed with a Zeta potential close to −38 mV. Transmission electron microscopy (TEM) results revealed the interior structure of the cluster of SPIO nanocrystals, with BSA formed the shell layer.

Additionally, it is imperative to assess the physical and chemical characteristics of nanosystem. The imaging capability of a contrast agent is a key factor in diagnosing illness. Herein, a 1.5 T MRI system and NIR-II fluorescence instrument were employed to analyze our nanosystem. As iron concentration of nanosystem rose from 0 to 0.37 mM, Figure 1C demonstrated that the MR-*T*_2_ weighted (T_2_WI) image signal decreased significantly, which was caused by the superparamagnetic property of iron oxide crystals in IR780@BSA@SPIO that reduced the transverse relaxation time of hydrogen nuclei in water and resulted in a dark color on the MR image. The correlation between the reciprocal *T*_2_ relaxation time and the concentration of IR780@BSA@SPIO was strong, with the fitting results revealing that the relaxation rate was 307.57 mM^−1^ s^−1^ based on the fitting results; and it was roughly three times greater than that of commercially available Feridex^@^ (63.8 nm, 98.4 mM^−1^ s^−1^) [28]. Consequently, the *T*_1_ relaxivity was about 2.00 /mM^−1^ s^−1^ (Supplementary Figure S2), leading to a *R*_2_ to *R*_1_ ratio that was far in excess of 20, making the nanosystem an outstanding *T*_2_ contrast agent. Additionally, IR780 molecule has a broad fluorescence emission range and maintains excellent imaging capabilities in the NIR-II region (>1000 nm) (Supplementary Figure S3) [29]. Therefore, the nanosystem demonstrated excellent fluorescence efficiency, and the signal brightness increased with the IR780 concentration (Figure 1D). The image on the top shows the fluorescence image of IR780@BSA while the one at the bottom displays the fluorescence image of IR780@BSA@SPIO. The analysis of the data revealed that the incorporation of SPIO nanocrystals did not significantly influence the fluorescence property of IR780 molecule. The combination of IR780@BSA@SPIO provides dual-modality imaging capabilities, which is advantageous for detecting lesions with high precision from MRI and guiding photothermal surgery via optical imaging.

It is thought that raising the temperature in the microenvironment of the tumor region will be able to eliminate the cancer cells, as the cancer cells have a low capacity to withstand heat [30]. Therefore, due to the photothermal effect of IR780 molecules, the absorption and heat swing dissipation experiment was conducted. In the Figure 1E, the highest temperature achieved in the heating process was 48°C at 1.5 μg/mL [IR780], and the four trials produced the same outcome; therefore, the photothermal efficiency of nanosystem showed a reliable and sustained effect. Moreover, it is widely acknowledged that cancer cells develop a special biochemical microenvironment with an acidic pH range, elevated H_2_O_2_, and reduced catalase activity [31]. Targeting these characteristics during therapy may reduce adverse effects and enhance the outcomes [32]. A prior investigation showed that SPIO nanocrystals had inherent peroxidase-like capability [33]. Therefore, when exposed to an acidic environment, the IR780@BSA@SPIO composite efficiently promoted the production of a hydroxyl radical from H_2_O_2_, resulting an UV–Vis absorption spectra of TMB oxidation with a peak at 652 nm. Additionally, the data suggested that light irradiation drastically amplified the catalytic reaction (Supplementary Figure S4), demonstrating its capability to create free radicals and improve therapeutic effect while reducing adverse reactions.

### Cellular imaging study and *in vitro* treatment with albumin-based nanosystem

It is desirable for nanomaterials to be compatible with living cells [34]. In this study, a CCK-8 kit was employed for cell proliferation and toxicity evaluation. The data in Supplementary Figure S5 indicated that, even when the concentration of IR780 was high (1.5 μg/mL, SPIO 13.6 μg/mL), IR780@BSA@SPIO exhibited minimal toxicity, and the cell activity in the experiment stayed above 80%. The satisfactory biocompatibility was sufficient to fulfill the demands of both *in vitro* and *in vivo* biological application.

**Figure 2.**
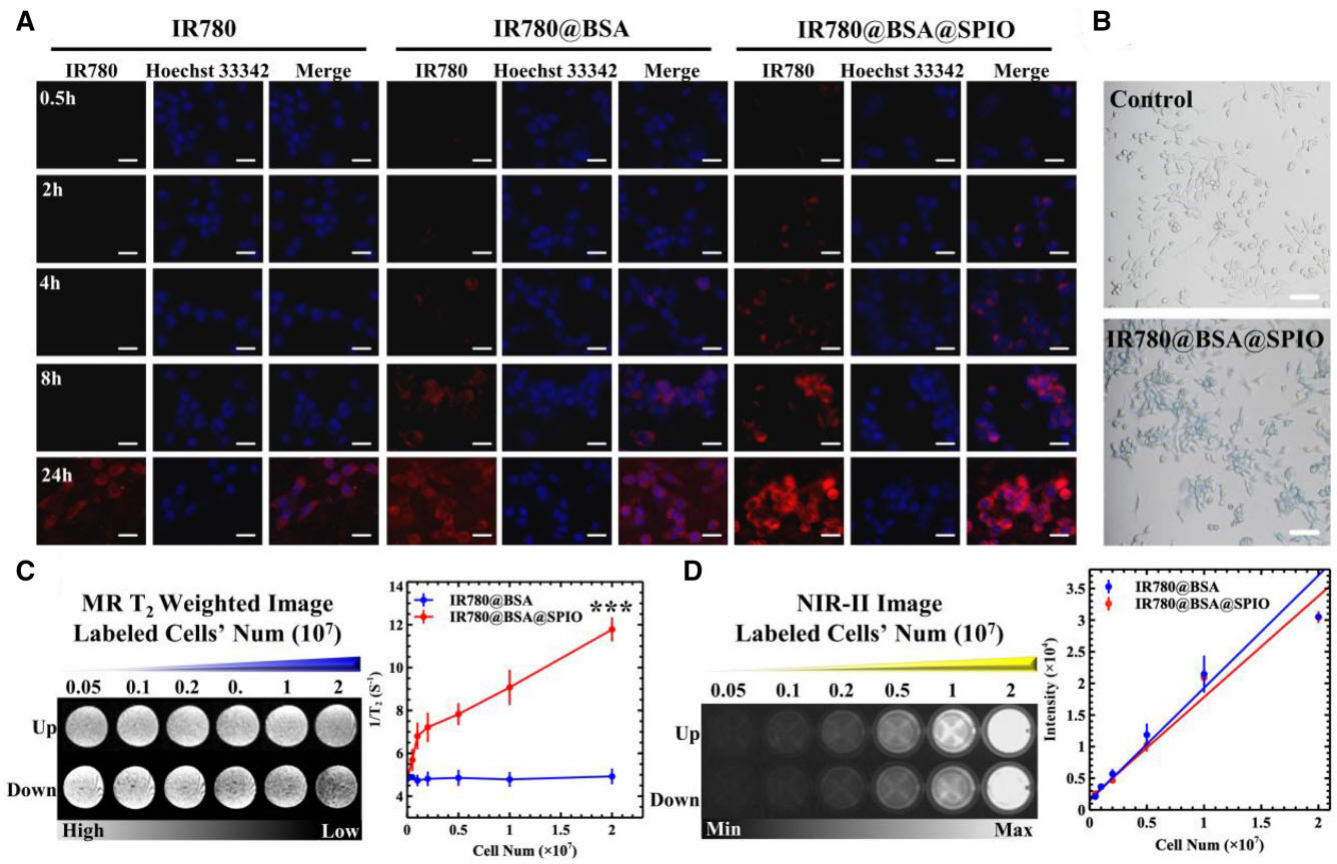
(**A**) Fluorescent image of 4T1 cells incubated with nanomedicines for different durations. Scale bar =100 μm. (**B**) Prussian blue staining of the nanosystem treated cells. Scale bar =500 μm. (**C**) MRI-*T*_2_ imaging of these labeled cells. And the relationship between the *T*_2_-relaxation time and the cell counts. (**D**) The fluorescence imaging of these labeled cells. And the relation curve between signal intensity and cells’ number. The ‘up’ and ‘down’ image refer to the fluorescence image of IR780@BSA and IR780@BSA@SPIO, respectively. *n* = 3, ***P < 0.001.

Some studies have demonstrated that cells can effectively take up nanoparticles with size between 50 and 70 nm [35]. To evaluate the capability of nanosystem’s cellular labeling and imaging results, some *in vitro* experiments were conducted. Herein, the nuclei were stained with Hoechst 33342, which was identified by its blue fluorescent signal, as indicated in Figure 2A. By increasing the incubation period of IR780@BSA@SPIO nanosystems and cells, the red dots started to appear in the cytoplasm, with a concentration around the nuclei, and these cells had almost 100% uptake after 24 h of treatment and emitted a strongest fluorescent signal comparing to all the control ones. In addition, a flow cytometry was used for quantitative analysis (Supplementary Figure S6) and the results demonstrated that the efficiency of drugs endocytosis was dependent on both the amount of time and the concentration. Interestingly, after 8 h of incubation, IR780@BSA@SPIO group had a greater uptake rate (96.5%) than IR780@BSA (59.2%) when the concentration of IR780 was 0.5 μg/mL. The uptake rate was increased close to 100% at [IR780] 1 μg/mL. Moreover, the Prussian blue staining method was used to measure the phagocytosis capability of cells toward nanodrugs, which was triggered by the presence of Fe_3_O_4_ crystals in the IR780@BSA@SPIO nanosystem [36]. The majority of cells demonstrated a blue hue upon staining, as demonstrated in Figure 2B, suggesting a greater uptake of the nanosystem when compared to the control ones.

Additionally, the imaging capability of the contrast agent permits the labeled cells to be efficient visualization and identification [37]. The intracellular fluorescence imaging effect from IR780@BSA@SPIO group increased in accordance with the number of cells, and the linearity of the fit was satisfactory, as seen in Figure 2D. Furthermore, Figure 2C illustrates the magnetic imaging of the labeled cells; the reciprocal relaxation time was roughly proportional to the number of cells, resulting in a darker T_2_WI image. The IR780@BSA control group, did not experience a significant *T*_2_ relaxation change effect or a noticeable alteration in weighted images. This suggests that the IR780@BSA@SPIO nanosystem was capable of shortening the transverse relaxation time of hydrogen protons in the intracellular environment, and when combining with MR imaging, this nanosystem could provide the necessary detection capabilities for investigating deeper tumors.

**Figure 3.**
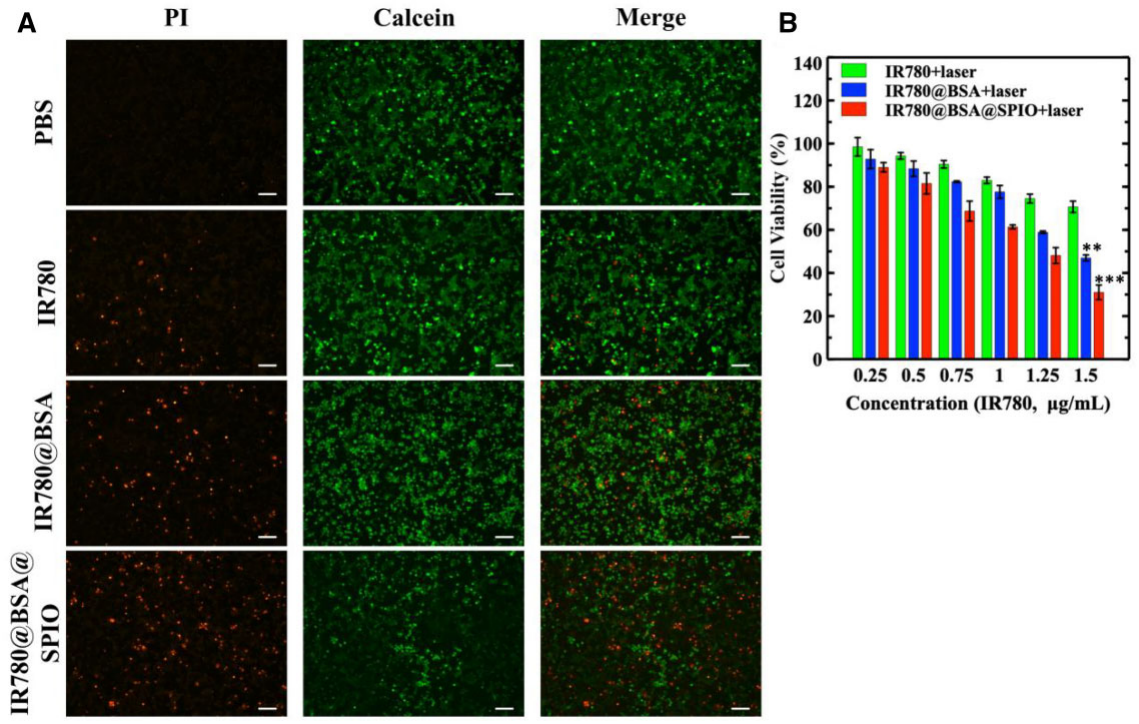
(**A**) AM/PI staining of different materials treated 4T1 cells after laser irradiation. (**B**) The cell viability of 4T1 cells after nanosystem-based phototherapy. The data are expressed as mean ± standard deviation (*n* = 3). Scale bar =800 μm. **P < 0.01, ***P < 0.001.

Hyperthermia, as a novel method for cancer therapy, has a bright future due to its convenient operation and controlled adverse effects [38]. Herein, the impact of *in vitro* hyperthermia on the system IR780@BSA@SPIO was investigated. The AM/PI staining image (Figure 3A) displayed green fluorescence from living cells and red signal from dead cells. The laser irradiation caused the red dots from PI dyes to be appeared in all three IR780 administrated groups, suggesting that the photo-treatment was responsible for the destruction of the 4T1 cell membrane. The therapeutic performance is also affected by the amount of drug, as is well known, and the photothermal killing effect was amplified as the intracellular IR780 concentration rises (Figure 3B). At the same time, in normal somatic cells, IR780@BSA@SPIO also showed a certain degree of photothermal lethality (Supplementary Figure S7). Therefore, these data demonstrated that the cells exposed to IR780@BSA@SPIO, with IR780 concentration about 1.5 μg/mL, had the highest number of red fluorescent spots, accounting for ∼70%; herein, the chemical catalysis property of the SPIO nanocrystals could also synergize with the light therapy, exerting a synergistic therapeutic effect that was more pronounced than the other groups. Thus, this novel nanosystem had been found a strong anti-tumor effect.

### *In vivo* dual-modality imaging for tumor diagnosis

**Figure 4.**
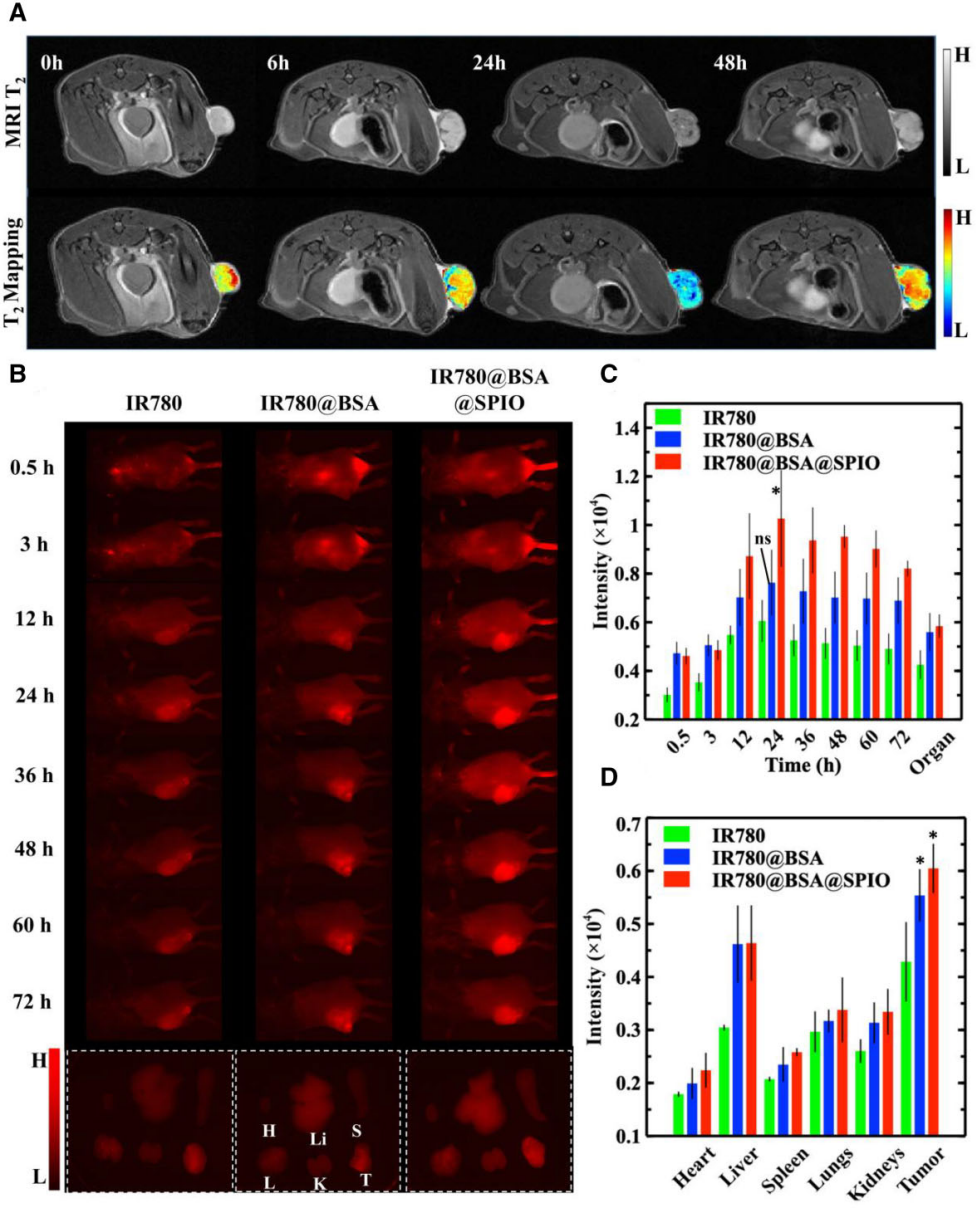
(**A**) *In vivo* MR imaging of tumor-bearing mice after injection of IR780@BSA@SPIO nanosystem. Color according to the local gray value. (**B**) NIR-II fluorescence imaging of tumor-bearing mice administrated with various drugs and isolated organs and tumors after 72 h (H: heart; Li: liver; S: spleen; L: lung; K: kidney; T: tumor). Semi-quantitative analysis of fluorescence intensity from tumor-bearing mice (**C**) and isolated organs and tumors (**D**). The data are expressed as mean ± standard deviation (*n* = 3). Signal intensity differences between BSA-based nanosystems and free IR780 group were analyzed, *P < 0.05.

Clinical imaging technology could effectively pinpoint the tumor, assess drug metabolism and optimal chem-phototherapy [39]. In this study, dual-modality imaging with the albumin nanosystem *in vivo* is used to guide the radiation of the verified lesion site and identify the optimal light time. In contrast to normal tissues, tumor present with abundant new blood vessels, inadequate structural stability, impaired lymphatic drainage and heightened permeability [40]. Thus, the nanoparticles could retention in tumor tissues owing to the EPR effect, thereby significantly increasing the delivery efficacy [41]. In the experiment, all the samples were injected into the tumor-bearing mice, respectively, followed by imaging detection. The clinical value of MRI lies in its ability to deliver detailed soft tissue information, and the contrast agent could further enhance its detection sensitivity [42]. Herein, the *T*_2_ signal intensity in the tumor areas was significantly decreased after 24 h of IR780@BSA@SPIO injection, compared to the signal before injection and the PBS group, which was markedly distinct from normal tissues, as demonstrated in Figure 4A and Supplementary Figure S8; and the fluorescence intensity of the tumor areas increased over time (Figure 4B and C), suggesting that the nanosystem was accumulating in the tumor areas. At 24 h after the injection of drug, IR780@BSA@SPIO group displayed the most potent magnetic resonance and fluorescence signal in the tumor region.

With metabolic alteration, the concentration of drugs in local areas will be modified, causing imaging information to fluctuate in time [43]. The data illustrated that MRI and fluorescence signals both demonstrated a recovery effect when the time extension was more than 24 h. However, at the end of the third day after administration, the fluorescent light from the tumor site was still intense. Subsequently, the mice were sacrificed and organs were harvested to measure the fluorescent intensity; as evidenced by the highest signal intensity at the tumor site (Figure 4B and D), demonstrating that this IR780@BSA@SPIO nanosystem with ∼50–70 nm was successful in performing the EPR effect. To conclude, the dual-modality imaging *in vivo* merges the benefits of both technologies, enabling accurate localization of tumor tissue, and pinpointing the optimal treatment time.

### *In vivo* photothermal therapy with the albumin-based nanosystem

**Figure 5.**
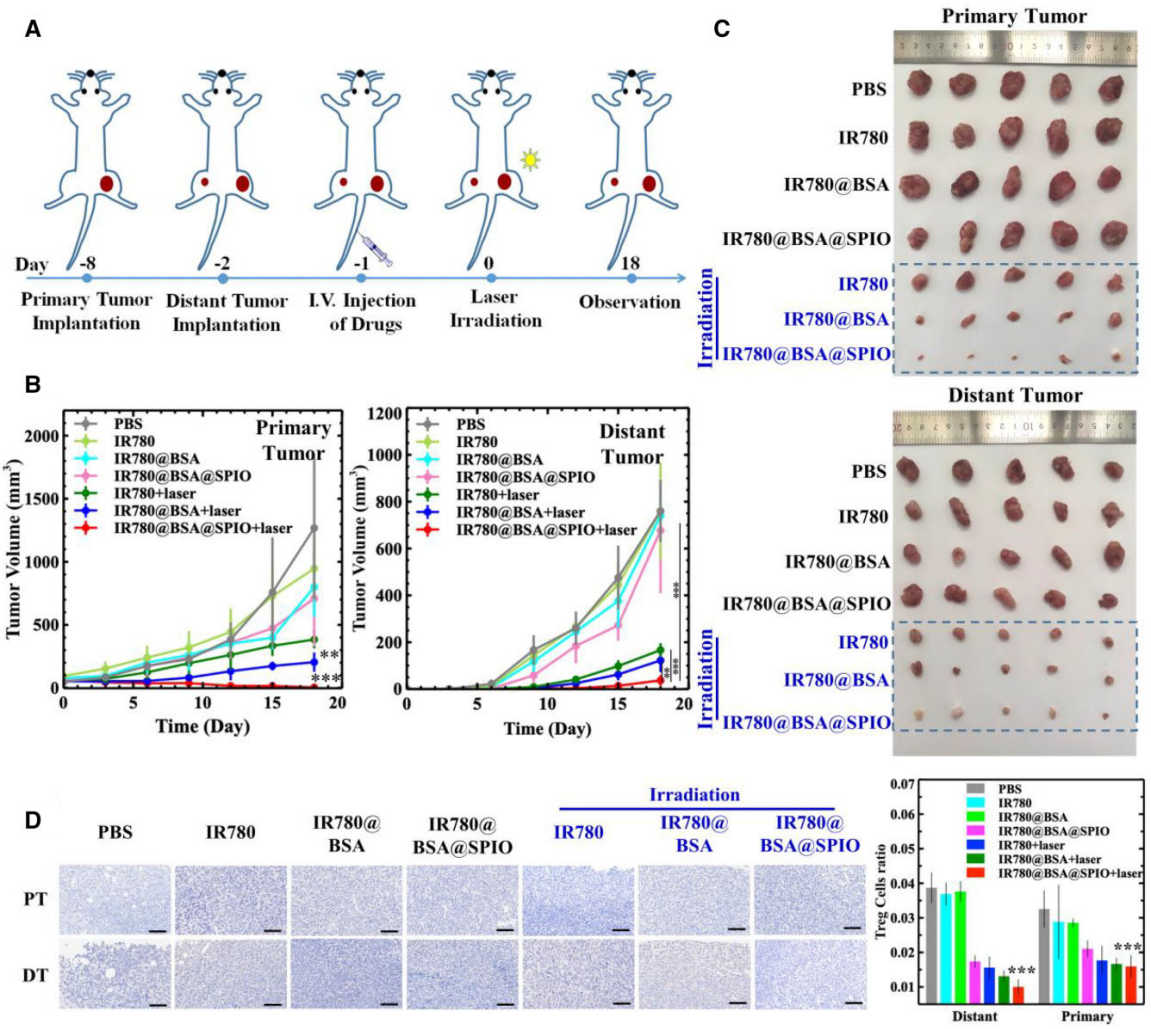
(**A**) Schematic illustration of the *in vivo* experiments. (**B**) Primary/distant tumor volume curve of tumor-bearing mice in different treatment groups. The data are expressed as mean ± standard deviation (*n* = 5). (**C**) Images of isolated primary and distant tumor from different treatment groups. (**D**) Foxp3 staining and semi-quantitative analysis of primary and distant tumor sections from different treatment groups. The data are expressed as mean ± standard deviation (*n* = 3). Scale bar =800 μm. The variation in tumor size between the IR780@BSA@SPIO nanosystem exposed to laser irradiation and the other groups was evaluated (**P < 0.01, ***P < 0.001).

The normal immune system usually monitors changes in biological information and respond accordingly, but the TNBC could suppress the immune response leading to more serious illnesses and metastasis [44]. Consequently, a metastatic TNBC model was established to assess the effectiveness of nanosystem-based therapy. Through MRI and NIR-II imaging guiding, photothermal therapy was performed on the primary tumor. The results from Supplementary Figure S8C demonstrated that the temperature of the tumor areas in the BSA-based two groups rose to 56°C after laser irradiation, which verified that the albumin system could increase the drug delivery capabilities as well as the photothermal stability of IR780. The tumor proliferation of the three groups of IR780 irradiation with laser were significantly hampered than those of the other groups, indicating the excellent photothermal therapeutical effects. The most successful therapeutical result was seen from the treatment group IR780@BSA@SPIO+laser (Figure 5B). The primary tumor shrank dramatically following a single radiation dose and nearly vanished after 18 days. Interestingly, the tumor situated distant from the radiation source showed a noteworthy reduction in growth, shrinking to a size of 36 mm^3^ over the course of 18 days of treatment, as opposed to the control group (PBS) that saw the volume of the primary tumor swell to 1000 mm^3^ and the volume of the distant tumor increase to 700 mm^3^ (Figure 5C). Conversely, the other three pure material groups without light (IR780, IR780@BSA and IR780@BSA@SPIO) failed to demonstrate any positive results for tumor therapy, and no significant difference was found between these groups.

### The activation of immunosystem with the albumin-based nanosystem

We hypothesized that the success of nano-drug therapy of this metastatic distant tumor was due to stimulation of the immune response and thus the activation of immune systems [45]. Therefore, the immune-related cells in the tumor areas were identified. Many studies have shown that there is generally a significant increase in the number of regulatory T cells (Tregs) in tumor tissue, and the close interaction between them and effector T cells exhibited an immunosuppressive and immune incompetent characteristic [46]. This kind of Tregs is indicated by a presence of Foxp3 [47]. Therefore, analyzing Foxp3 staining in both primary tumor and distant tumor sections could reveal treatment outcome. Herein, the number of Treg cells in the irradiation-based treatment groups and the free IR780@BSA@SPIO group was significantly lower than the others, demonstrating that the combination of laser irradiation and BSA nanosystem can successfully decrease the amount of Tregs, thus eliciting a host immune response and facilitating tumor treatment (Figure 5D).

**Figure 6.**
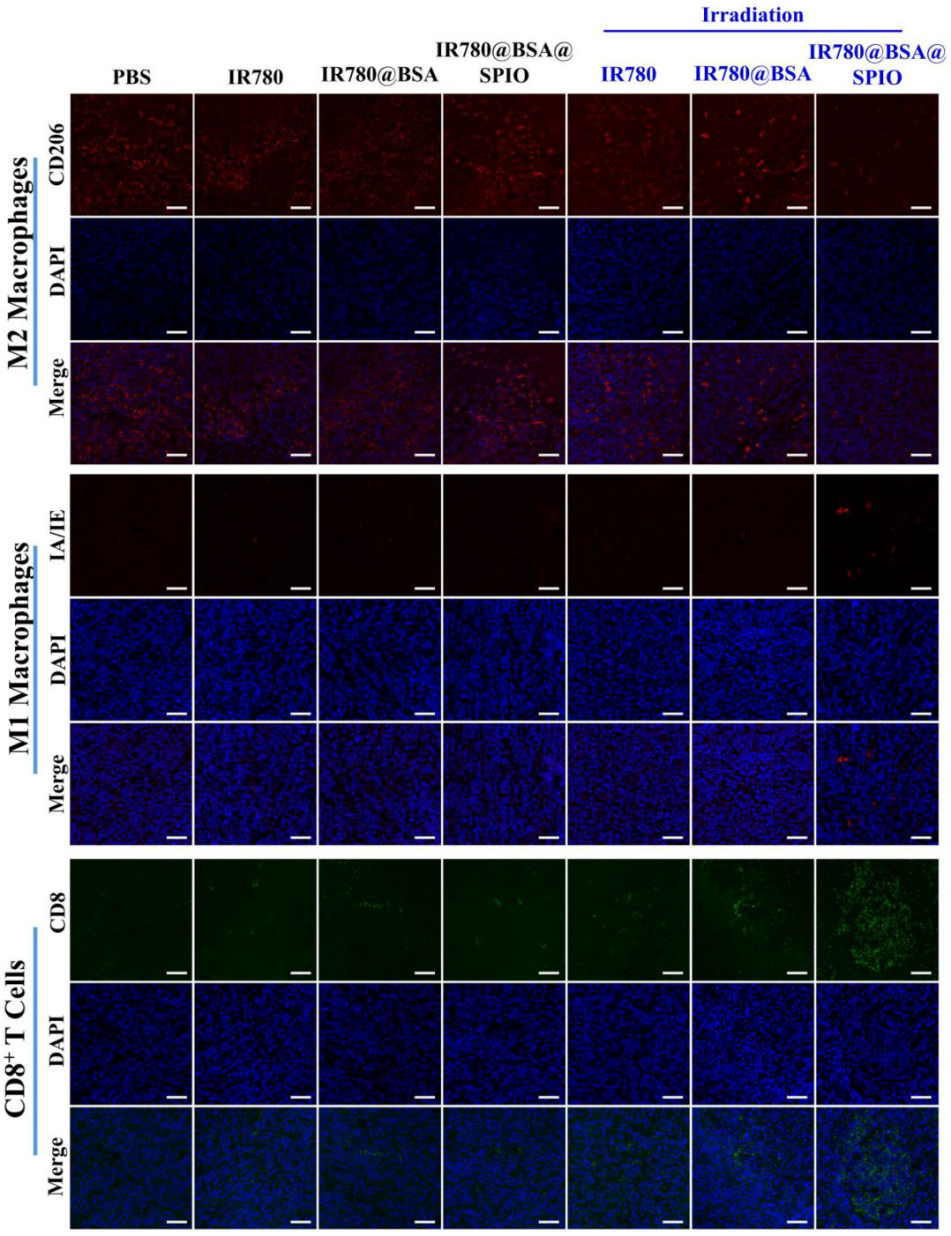
Immunofluorescence imaging of primary tumor sections in different treatment groups. Scale bar =800 μm.

It is well established that macrophages can be divided into two types, namely the M1 and M2 types, according to their activation status and function [48]. Herein, the M1 type macrophages perform proinflammatory responses; however, phenotypic switch of M1 into M2 macrophage in tumor microenvironment shows an immunosuppressive effect and thus aids tumor progression [49]. Figure 6 displays the results of staining tumor tissue sections, and the biomarkers CD206 (red) and MHC II (IA/IE, red) were used to identify the cell phenotype. It showed a reduction of M2 macrophages labeled with red fluorescence and an augmentation of M1 macrophages labeled with green fluorescence dyes in the IR780@BSA@SPIO+laser treated group, which indicated that the photothermal treatment was successful in stimulating the polarization of macrophages in tumor tissue from M2 to M1. Moreover, the CD8^+^ T cells as an integral part of the immune system are capable of recognizing and responding to antigens on the surface of tumor cells with the help from M1 macrophages, allowing them to attack cancers directly [50]. In Figure 6, the results showed that the IR780@BSA@SPIO group underwent light irradiation had a higher concentration of CD8^+^ T cells in the vicinity of the tumor tissue, compared to other control ones. This indicates the nanosystem may be a viable approach for stimulating an immune response to the tumor. And then, we also tracked the level of cytokines interferon-γ and tumor necrosis factor-α, two prominent proinflammatory cytokines (Supplementary Figure S9). The mice that underwent combined photothermal and catalytic therapy exhibited increased concentrations of two immune factors in comparison to the control groups, which demonstrated the success of the treatment in activating the immune system and enhancing the anti-tumor immune response.

**Figure 7.**
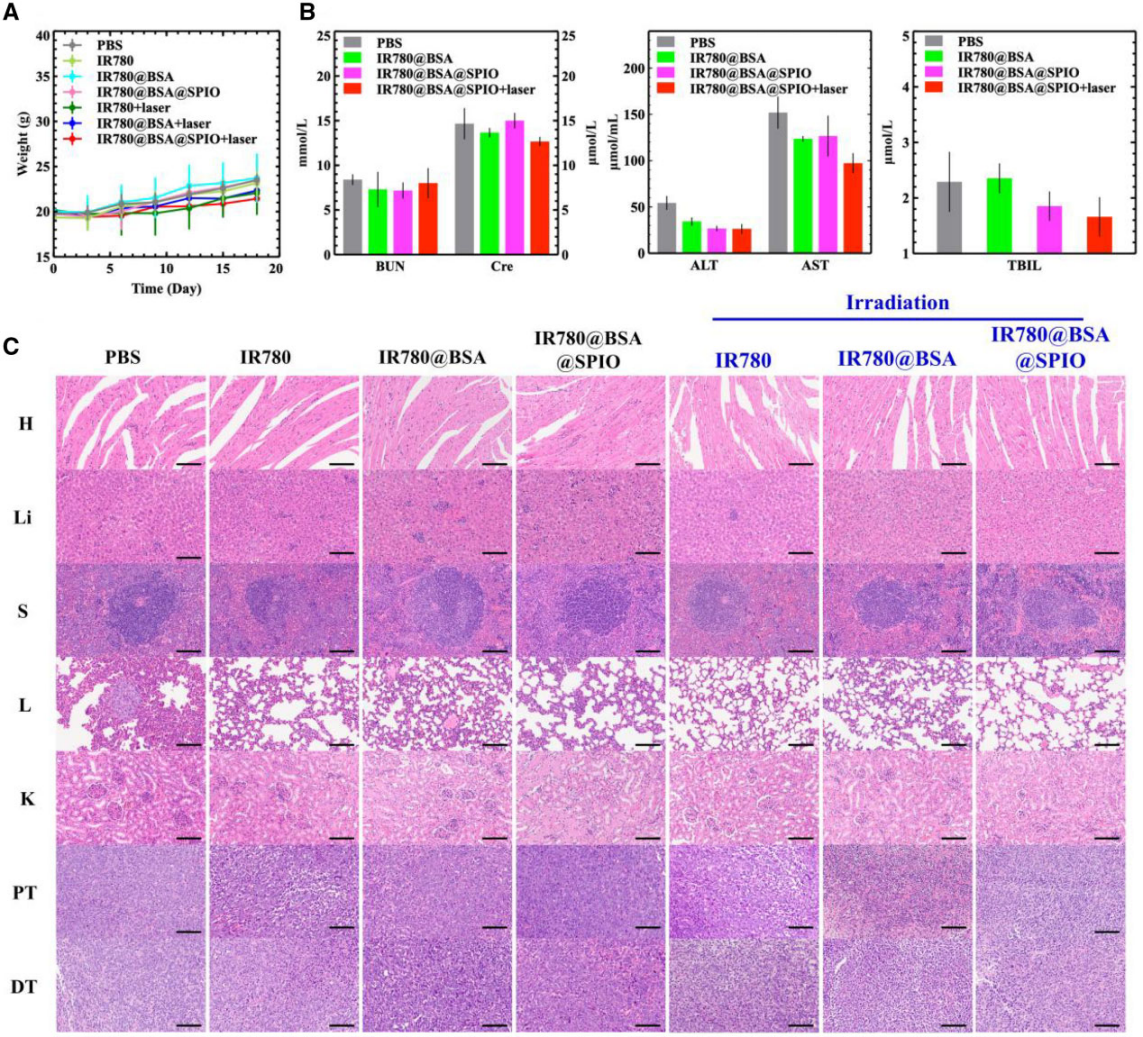
(**A**) Body weight curves of tumor-bearing mice in different treatment groups. The data are expressed as mean ± standard deviation (*n* = 5). (**B**) Biochemical analysis of blood components: ALT, AST, BUN, Cre and TBIL. The data are expressed as mean ± standard deviation (*n* = 3). (**C**) Hematoxylin and eosin staining of main organs (H: heart; Li: liver; S: spleen; L: lung; K: kidney), primary tumor and distant tumor sections of tumor-bearing mice after treatment. Scale bar =800 μm.

At the same time, we also monitoring physiological indicators of the mice during treatment, and the results indicated that the drug alone and the photoirradiation group did not show considerable adverse effects on the animals’ weight gain relative to the untreated group (PBS group) (Figure 7A). Furthermore, following the experiment, the serum biochemical indicators of the mice in each group demonstrated that the alanine aminotransferase (ALT), aspartate aminotransferase (AST), total bilirubin (TBIL), blood urea nitrogen (BUN) and creatinine (Cre) indicators of the IR780@BSA@SPIO combined photothermal treated group were comparable and marginally lower than the control one (Figure 7B). Furthermore, the analysis of the major organs showed that nanomaterial-mediated therapy did not have adverse effects (Figure 7C) suggesting that the treatment was highly biocompatibility and biosafety.

## Conclusion

The aggressive nature of TNBC, with its tendency to metastasize and recurrence, makes it difficult to accurately diagnose and therapy [51]. This study developed a multifunctional nanosystem, IR780@BSA@SPIO with the capability to perform NIR-II and MR dual-modality imaging, enzyme-like catalytic and photothermal conversion. Through this, the imaging technology could be used to accurately identify and locate lesions to provide assistance to light irradiation. Subsequently, the therapy not only facilitates the efficient eradication of tumors in their original place but also activates and stimulates the immune system was also to impede the growth of metastatic tumors, which is even more importantly. The therapy procedure exhibits an excellent biocompatibility and biosecurity. Therefore, this nano-drug-based therapy could prove to be highly beneficial in strengthening the immune system and impeding the proliferation and reoccurrence of malignancy.

## Materials and methods

### Materials and reagents

McLean, China was the source of Iron(III) acetylacetonate, 1,2-hexanediol, oleamine and benzyl ether. Niacin and BSA were bought from China’s Aladdin. IR780 dyes and oleic acid were from Sigma-Aldrich, USA. Local companies supplied other chemical reagents. Mouse breast cancer 4T1 cell line was obtained from the American Type Culture Collection. Trypsin EDTA solution (0.25%), fetal bovine serum and penicillin streptomycin were purchased from Gibco, USA. Calcein-AM/PI Staining Kit and Cell Counting Kit-8 were bought from Biyuntian Biotechnology Co., Ltd, China. Hoechst 33342 and Perls dye were purchased from Solarbio Technology Co., Ltd, China. ELISA MAXTM Deluxe Set Mouse TNF-α and ELISA MAXTM Deluxe Set Mouse IFN-γ were purchased from BioLegend, USA. Animal experiments were approved by the Laboratory Animal Management Committee of Sun Yat-sen University.

### Preparation and characterization of albumin nanosystem

The CMC of nanoparticles was measured by using a fluorescence spectrophotometer (F-7000, Hitachi High-Tech, Japan) to calculate the ratio of fluorescence intensities at 338 and 333 nm from the pyrene fluorescence probe.

SPIO nanocrystals were prepared via high-temperature solvent thermal decomposition method [52], and characterized through TEM (JEM-1400 Plus, JEOL, Japan) and dynamic light scattering (DLS) (NanoBrook Omni, USA). IR780@BSA@SPIO was prepared by self-assembly method. Firstly, 1 mg IR780, 10 mg niacin, 10 mg dimethylimidazole and 20 mg SPIO nanocrystals were dissolved in 500 μL trichloromethane; then, the mixture solution was added into 5 mL deionized water containing 10 mg BSA and kept sonicating for 1 min. The IR780@BSA@SPIO solution was obtained by rotating and evaporating the residual THF solvent and dialysis. The size, Zeta potential and morphology of IR780@BSA@SPIO nanosystem were characterized by DLS and TEM. The concentration of IR780 and iron were measured through UV–Vis spectrophotometer (UV-2600, Shimadzu, Japan) and ICP (TJA IRIS-HR, USA), respectively, to calculate the encapsulation efficiency. The 1.5 T magnetic resonance system (HT-MICNMR-60, Shanghai Huantong Science and Education Equipment Co., Ltd, China) was applied to analyze the *T*_2_ relaxation time of nanosystem. Herein, the parameters were as follows: T_2_WI, repetition time (TR): 10 000 ms, echo time (TE): 7.73–800 ms; slice thickness: 3 mm. The UV–Vis absorption and photoluminescence spectra of the free IR780 and IR780@BSA@SPIO were measured through UV–Vis spectrophotometer (UV-2600, Shimadzu, Japan) and Fluorescence spectrometer (FLS1000, Edinburgh, UK) with the Ex at 780 nm. The photothermal effect was verified by irradiation with a 1 W/cm^2^, 808 nm laser and a certain gradient IR780 concentration, and then, the real-time temperature was recorded every 30 s. The stability of the photothermal effect of the material was analyzed by heat absorption and dissipation rocking experiment; herein, after 5 min of laser exposure, take a break for 5 min and repeat this cycle four times.

The substrate TMB was employ to assess the catalytic capability of IR780@BSA@SPIO nanosystem, which manifested as a transition from colorless to blue. In this experiment, a solution (500 μL, pH = 6.5) containing IR780@BSA@SPIO (5 μg), H_2_O_2_ (50 μmol) and 5 μL TMB (0.25 μmol) were mixed and incubated at 50°C for 10 min. Subsequently, the absorbance at 652 nm was determined using a UV–Vis spectrophotometer. To further explore the photochemical impact of IR780@BSA@SPIO on its catalytic performance, the nanosystem solution was irradiated under 808 nm laser (1 W/cm^2^) for 1 min, and the absorption peak was measured after 10 min.

### Cell culture of albumin nanosystem *in vitro*

To assess cytotoxicity, 4T1 cells (1 × 10^4^ cells per well, 96-well) were cultured in RPMI-1640 medium containing free IR780, IR780@BSA and IR780@BSA@SPIO, respectively, for 24 h. Then, CCK-8 assay was used to detect the cell activity with Infinite M200 PRO NanoOuant microplate reader (Tecan, Switzerland).

To test the cell uptake efficiency of drugs, 4T1 cells were cultured in IR780@BSA@SPIO medium containing 1.5 μg/mL IR780 for 24 h, and then stained with Russian blue. In addition, 4T1 cells were treated with different drugs (free IR780, IR780@BSA and IR780@BSA@SPIO) (IR780, 0.5 μg/mL), stained with Hoechst 33342, and observed the drug uptake efficiency with inverted fluorescent microscope (BZ-X800, Keens Co., Ltd, China). The 4T1 cells (1 × 10^6^ cells per well, 12-well) cultured with IR780@BSA or IR780@BSA@SPIO (IR780, 1.5 μg/mL) was also measured using flow cytometry (APC Alexa Fluorchannel, BD Biosciences, USA). To test the property of labeled cells’ MRI *T*_2_ relaxation and fluorescence imaging effect, the cell suspension was prepared according to a certain concentration gradient, mixed with 1% agarose gel and detected through the 1.5 T MR system and NIR-II fluorescence *in vivo* imaging system with a 75-ms exposure time (*in vivo* master, Wuhan Guangyingmei Technology Co., Ltd, China).

4T1 cells (1 × 10^4^ cells per well) were cultured in RPMI-1640 medium containing free IR780, IR780@BSA and IR780@BSA@SPIO, respectively. And then, we irradiated each well with a 1 W/cm^2^, 808 nm laser for 5 min; afterwards, the cell survival rates were measured using the CCK-8 method. In addition, the cells were also stained with Calcein-AM/propidium iodide, and the survival/death cells were observed under an inverted fluorescent microscope.

### *In vivo* MRI and NIR-II dual-modality imaging of albumin nanosystem

Balb/c mice (7 weeks old, female) were selected, and 2 × 10^6^ 4T1 cells were injected to each mouse subcutaneously to establish a breast cancer model. The mice carrying tumors were randomly split into three groups. Free IR780, IR780@BSA and IR780@BSA@SPIO (200 μL, 2.5 μg/mL IR780, 46.25 μg/mL SPIO) were respectively injected into tail vein of mice in each group. The NIR-II fluorescence *in vivo* imaging system was carried out and a 9.4 T MRI instrument (Bruker, America) was used to perform *in vivo* MRI studies. Before the injections and 6, 24 and 48 h post-injection, the T_2_WI and *T*_2_ mapping images were obtained; herein, the parameters were as follows: T_2_WI, TR: 2000 ms, TE: 20 ms; slice thickness/gap: 0.7/1.0 mm; and matrix: 192 × 256. After 72 h, the mice were sacrificed and key organs were separated for imaging analysis.

### *In vivo* therapy with albumin nanosystem

To establish a TNBC model, 2 × 10^6^ 4T1 cells were injected to Balb/c mouse on the back of the right hind leg. Seven days later, 2 × 10^6^ 4T1 cells were used to construct distal and metastatic tumors on the back of the left hind leg subcutaneously. The tumor-bearing mice were divided into seven groups randomly: Group 1, injected PBS (200 μL); Group 2, injected free IR780 (200 μL, 20 μg/mL, IR780); Group 3, injected IR780@BSA (200 μL, 20 μg/mL, IR780); Group 4, injected IR780@BSA@SPIO (200 μL, 20 μg/mL, IR780); Group 5, injected free IR780 (200 μL, 20 μg/mL, IR780) and irradiated the right tumor site with laser on the next day; Group 6, injected IR780@BSA (200 μL, 20 μg/mL, IR780) and irradiated the right tumor site on the next day; Group 7, injected IR780@BSA@SPIO (200 μL, 20 μg/mL, IR780) and irradiated the right tumor site on the next day. Each mouse was irradiated with 1 W/cm^2^, 808 nm laser for 10 min. Real-time tumor temperature was recorded. At the same time, the body weights and tumor volumes of these mice were monitored every 3 days. The mouse blood was collected on the 0, 6, 12 and 18 days for TNF-α and IFN-γ immune factor detection. After 10 days, the tumor and main organs were taken, and tissue sections were prepared. Hematoxylin–eosin staining was used for histological monitoring. Foxp3 immunohistochemical staining was also performed on the tumor to calculate the proportion of Treg cells.

To test the biocompatibility, after 18 days of treatments, some mice were sacrificed and serum was obtained to monitor liver and kidney functions. The specific indicators of BUN, Cre, ALT, AST and total TBIL were observed.

### Statistical analysis

All statistical analyses were performed using the software Veusz 3.3.1. Single factor analysis of variance and test were used for difference analysis. The data were analyzed and expressed in the form of mean ± standard deviation. (*P < 0.05, **P < 0.01, ***P < 0.001).

## Supplementary Material

rbad073_Supplementary_Data

## Data Availability

Data will be made available on request.
