# Supplementary material for: Albumin-based nanosystem for dual-modality imaging-guided chem-phototherapy against immune-cold triple-negative breast cancer
Source: Regen Biomater. 2023 Aug 31;10:rbad073. doi: 10.1093/rb/rbad073 (PMC10548782; doi:10.1093/rb/rbad073)
Supplement: rbad073_Supplementary_Data [file rbad073_supplementary_data.pdf]

Support Information

Albumin based nanosystem for dual-modality imaging guided chem-phototherapy against immune cold triple-negative breast cancer

Chen Peng [a,1], Xiaodie Zeng [a,1], Jiali Cai [a,1], Hanyu Huang [a], Fang Yang[d], Shaowen Jin[c***], Xiuhong Guan [b **], Zhiyong Wang [a*]

a. Key Laboratory for Polymeric Composite and Functional Materials of Ministry of Education, School of Materials Science and Engineering, Sun Yat-Sen University, Guangzhou, 510275, China

b. Department of Radiology, The Sixth Affiliated Hospital of Guangzhou Medical University, Qingyuan People's Hospital, Qingyuan, 511518, China

c. Department of Gastrointestinal Surgery, Sun Yat-Sen Memorial Hospital, Sun Yat-Sen University, Guangzhou, 510120, China

d. Department of Pediatrics, Department of Nuclear Medicine, Guangdong Provincial Engineering Research Center of Molecular Imaging, the Fifth Affiliated Hospital, Sun Yat-sen University, Zhuhai, 519000, China

1 The authors contributed equally to this work.

Corresponding authors

Zhiyong Wang: wangzhiy3@mail.sysu. edu.cn

Xiuhong Guan: xiuhongguan20@163.com

Shaowen Jin: jinshw3@mail.sysu.edu.cn

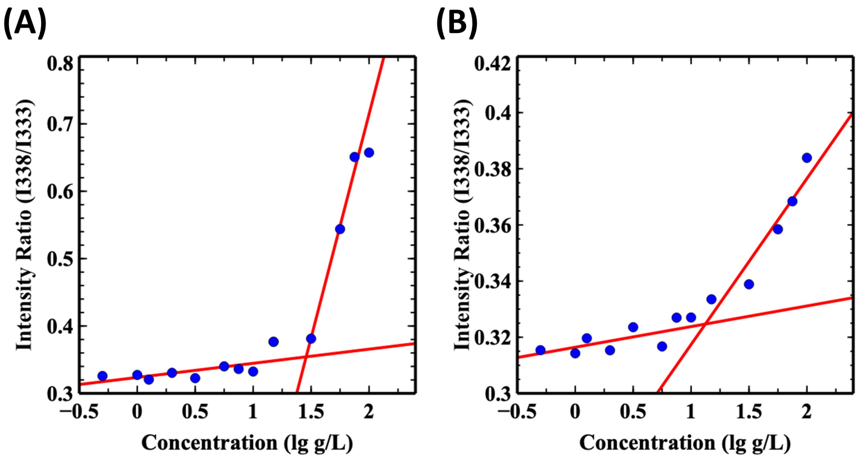

Fig. S1 Critical micelle concentration of (A) BSA and (B) BSA based nanosystem by pyrene fluorescence probe method (I338/I333).

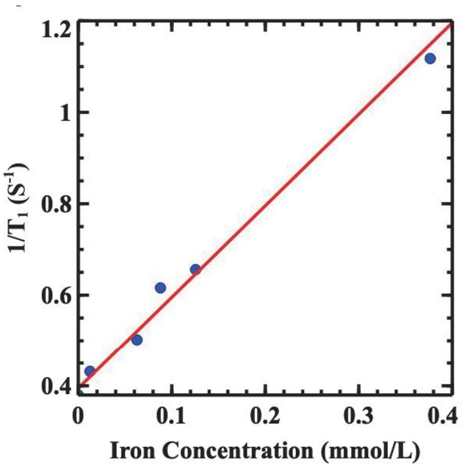

Fig. S2 Relationship between MR longitudinal relaxation time and concentration of IR780@BSA@SPIO nanosystem.

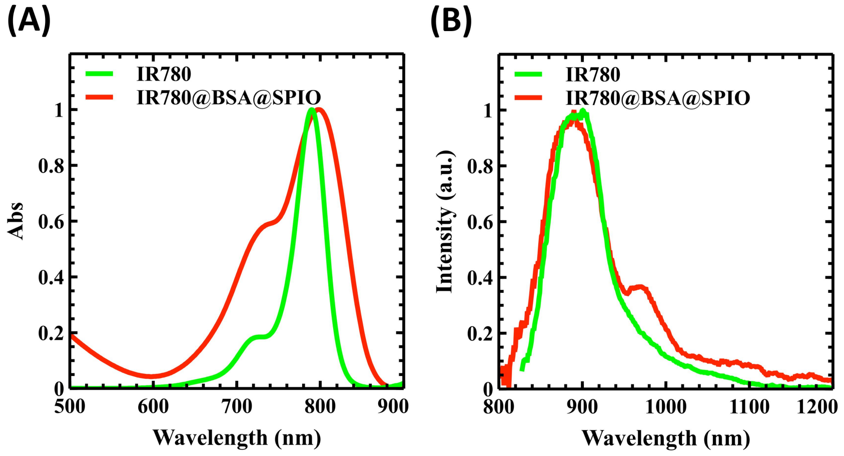

Fig. S3 UV-vis absorption (A) and photoluminescence spectra (B) of the free IR780 and IR780@BSA@SPIO.

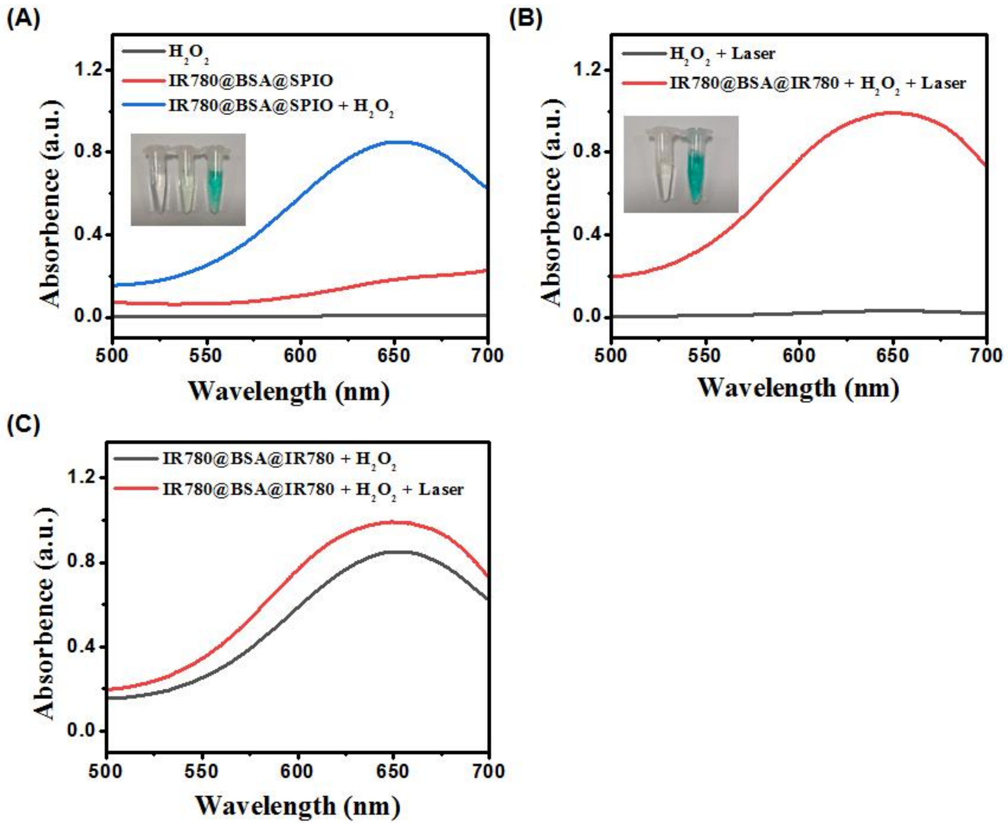

Fig. S4 The UV-Vis spectrum and photos of the oxidized TMB from the reaction of IR780@BSA@SPIO nanosystem with the H_2O_2 under different conditions.

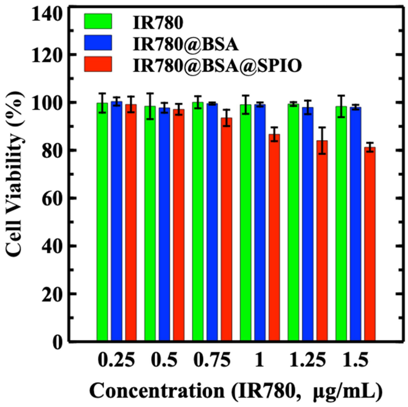

Fig. S5 The cytotoxicity of the material towards 4T1 cells at different concentrations. The data are expressed as mean ± standard deviation (n=3).

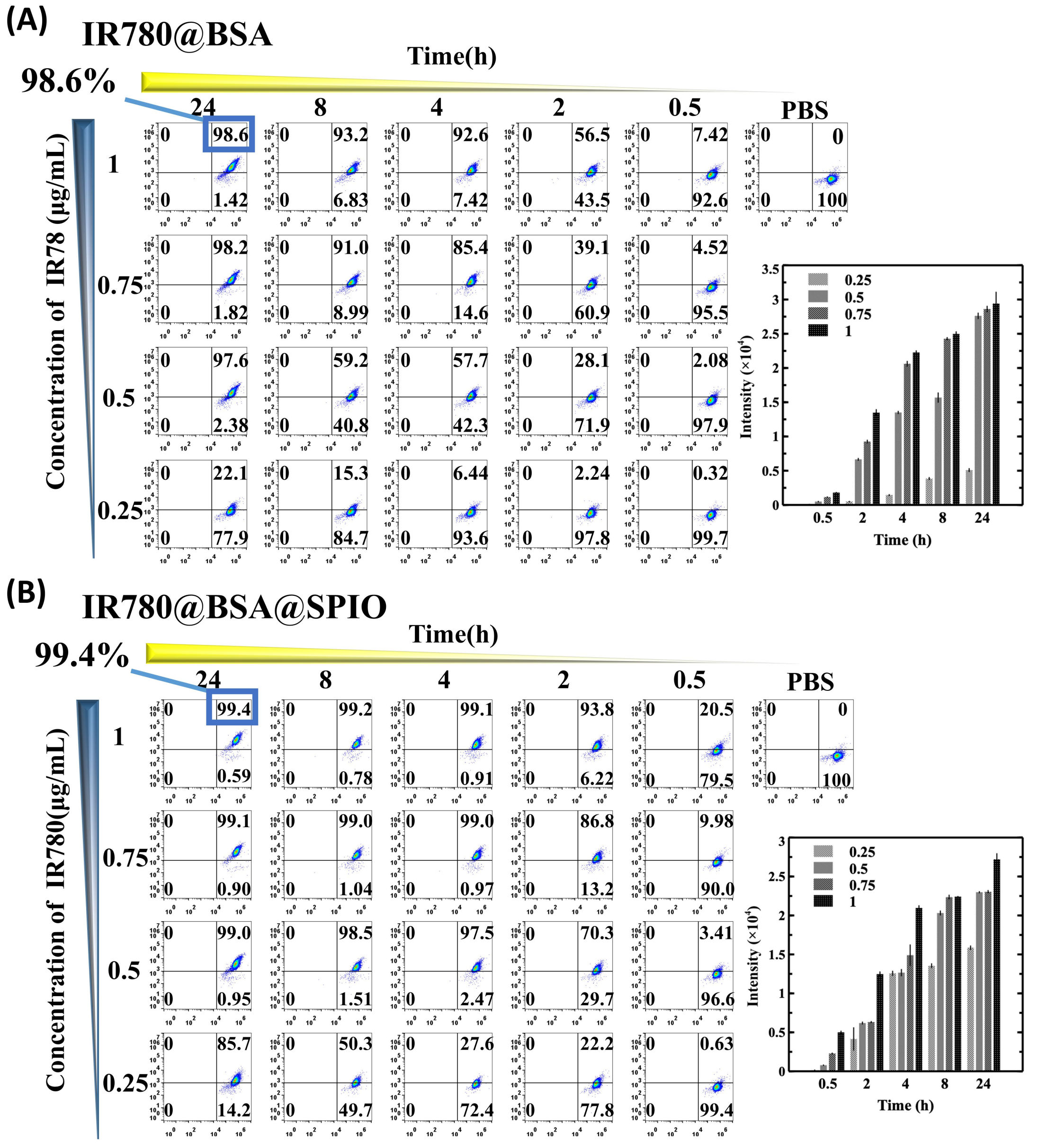

Fig. S6 Flow cytometry was used to quantitatively analyze the IR780@BSA (A) and IR780@BSA@SPIO (B) nanodrugs uptake effect of 4T1 cells at different drug concentrations and incubation times. The data are expressed as mean ± standard deviation (n = 3).

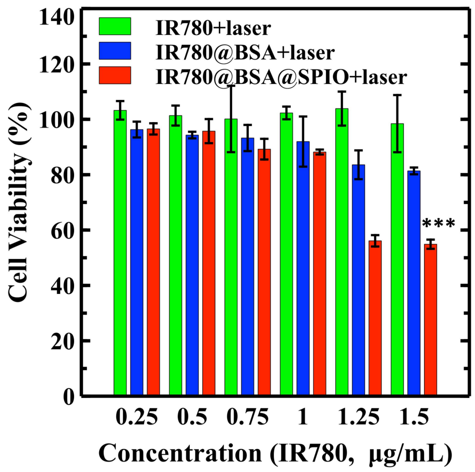

Fig. S7 The cell viability of 293T cells after nanosystem based phototherapy. The data are expressed as mean ± standard deviation (n = 3). ***$P < 0.001$.

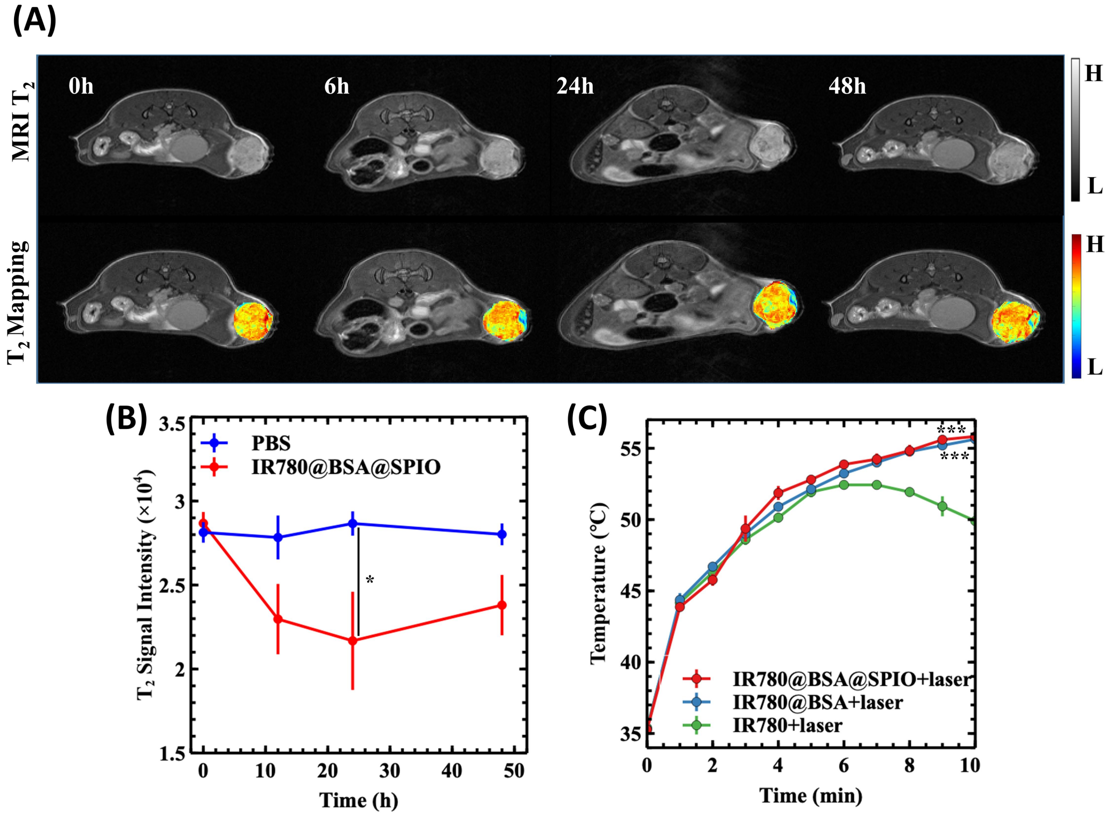

Fig. S8 (A) *In vivo* MR imaging analysis of tumor-bearing mice after PBS injection into the tail vein. Color according to the local gray value. (B) Semi-quantitative MR imaging analysis of tumor-bearing mice after administration with PBS and IR780@BSA@SPIO nanosystem. The data between two groups is evaluated. (C) Temperature changes in the tumor area in different treatment groups. The data are expressed as mean ± standard deviation (n = 3). *P < 0.05, ***P < 0.001.

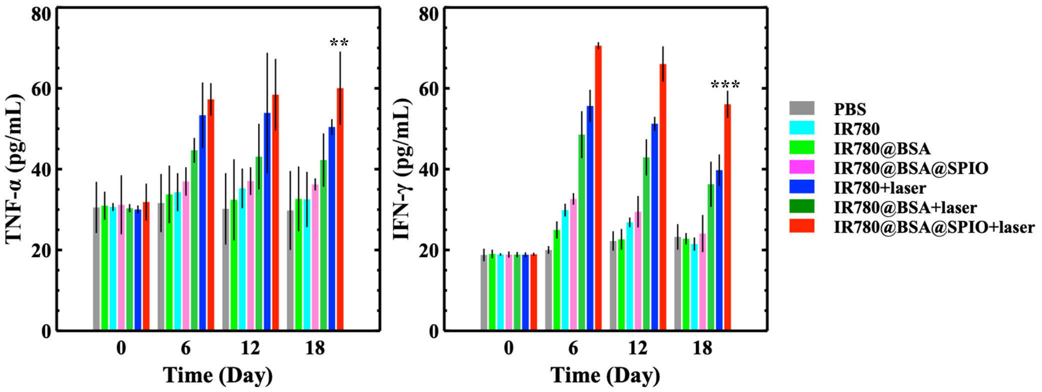

Fig. S9 The levels of TNF-α and IFN-γ inflammatory cytokines from different treatment groups. The data between the IR780@BSA@SPIO nanosystem exposed to laser irradiation and the other groups are evaluated and expressed as mean ± standard deviation (n = 3). $^{**}P < 0.01$, $^{***}P < 0.001$.
